# Modeling the Effects of Moisture-Related Skin-Support Friction on the Risk for Superficial Pressure Ulcers during Patient Repositioning in Bed

**DOI:** 10.3389/fbioe.2013.00009

**Published:** 2013-10-14

**Authors:** Eliav Shaked, Amit Gefen

**Affiliations:** ^1^Department of Biomedical Engineering, Faculty of Engineering, Tel Aviv University, Tel Aviv, Israel

**Keywords:** microclimate, finite element analysis, tissue breakdown, wetness, incontinence

## Abstract

Patient repositioning when the skin is moist, e.g., due to sweat or urine may cause skin breakdown since wetness increases the skin-support coefficient of friction (COF) and hence also the shear stresses that are generated in the skin when the patient is being moved. This everyday hospital scenario was never studied systematically however. The aim of this study was to simulate such interactions using a biomechanical computational model which is the first of its kind, in order to quantitatively describe the effects of repositioning on the pathomechanics of moisture-related tissue damage. We designed a finite element model to analyze skin stresses under a weight-bearing bony prominence while this region of interest slides frictionally over the support surface, as occurs during repositioning. Our results show, expectedly, that maximal effective stresses in the skin increase as the moisture-contents-related COF between the skin and the mattress rises. Interestingly however, the rise in stresses for a wet interface became more prominent when the skin tissue was stiffer – which represented aging or diabetes. This finding demonstrates how the aged/diabetic skin is more fragile than a young-adult skin when repositioning in a moist environment. The modeling used herein can now be extended to test effects of different moisturizers, creams, lubricants, or possibly other interventions at the skin-support interface for testing their potential in protecting the skin from superficial pressure ulcers in a standard, objective, and quantitative manner.

## Introduction

There is a debate over the current classification of pressure ulcers (PUs), as the recent literature indicates continuous challenges with respect to the physician’s consensus and conventional thinking. There are questions regarding the validity of definitions of PUs in general, mainly over the place of superficial PUs (SPUs) in the current classification provided by the European Pressure Ulcer Advisory Panel (EPUAP) (Houwing et al., 2007; Lahmann and Kottner, 2011; Lahmann et al., 2011). Revisiting classifications, may result in a substantial economic impact and major burden for current healthcare; however, it may improve the preventability rate of PUs (Reddy et al., 2008). In addition, this retrospective approach provides new clinical prospects for management and treatment of PUs that may improve pressure ulcer understanding and hence prevention, as it opens a fresh glance to the pathomechanics of tissue damage that manifests as SPUs (Gefen, 2011).

The EPUAP defines PUs as “an area of localized damage to the skin and underlying tissue caused by pressure, shear, friction, or a combination of these” and their classification system is summarized by four grades (grade I–IV) that indicate the severity of the wound (Houwing et al., 2007). Practically, SPUs correspond to Grade I and Grade II PUs (Gefen, 2011).

Recent research proposes modification predominantly based on the distinct mechanisms of PUs: superficial skin changes occur from the outside in, and deep PUs from the inside out (Sibbald et al., 2011). Therefore, the current concepts of grades could be falsely defined – based on lack of understanding of the etiology. In particular, the etiology of SPUs likely involves interacting thermodynamic and mechanical factors at the microenvironment of the skin (often being termed “microclimate”), which in turn affects friction, internal loading in skin and subcutaneous tissues and their failure thresholds (strength properties). In an attempt to examine some microclimate factors and their impact on SPUs, we recently developed a mathematical model that supports empirical findings and clinical observations concerning risk factors and risk assessment for SPUs (Gefen, 2011). Our model concludes that changes in the microclimate of the skin, which includes the local temperature and moisture conditions, on and around the skin, at weight-bearing regions of the body, involve risk for SPUs. Quantitatively, the model demonstrated that increases in skin temperature, ambient temperature, relative humidity, pressure delivered to the skin from the support and decreased permeability of the support materials in contact with the skin or in close proximity to the skin – all raise the risk for SPUs (Gefen, 2011). The most prone anatomical regions to SPUs for bed-bound patients is skin near bony prominences (Edwards and Marks, 1995; Hendriks and Franklin, 2010).

For immobile and bed-bound patients, the tolerance of skin is constantly challenged by factors affecting from the outside in, e.g., frictional forces at the skin surface, that changes due to microclimate conditions, clothing and bed sheet materials, interface pressures, relative motion and sliding velocity, as well as a moist or wet skin, e.g., due to perspiration or incontinence (Gerhardt et al., 2008b; Derler and Gerhardt, 2011). Moreover, these parameters influence deeper tissue layers, as there are physical and biomechanical interactions between the skin and deeper tissues (Kottner et al., 2011).

The literature identifies a relationship between wet skin, particularly due to trapped perspiration and incontinence, and an increased risk for SPUs (Cakmak et al., 2009), and also mentions that the risk increases further if the exposure is to urine as opposed to just water. This has been quantified for example in a human study where pads saturated with water and with a water solution mixed with the main chemical constituents of urine (synthetic urine) were applied to forearm skin of healthy subjects for 5.5 h (Mayrovitz and Sims, 2001). The researchers found that synthetic urine and water reduced the skin hardness and perfusion during pressure loads when compared with dry sites, however, the study was focused on static loading. In patient populations, those suffering occasional incontinence are also at risk for more severe PUs (van Rijswijk, 1993), but it is extremely difficult to isolate just the effect of the incontinence on the risk for PUs in real-world scenarios, where there are typically several co-morbidities and many potential risk factors. Theoretical modeling is therefore needed in order to complement these subject studies and better identify the mechanisms and underlying factors in the cascades that cause skin failure. The present work starts this modeling effort by simulating repositioning of a body area prone to PUs in a wet environment.

The work of Vanderwee et al. (2007) on repositioning is perhaps the most cited modern literature with respect to the need for moving and turning patients in bed in order to protect them from PUs. One of their points of focus was the frequency at which these repositioning interventions would be most effective with respect to cost of nursing manpower. They have looked at patients lying 2 h in a lateral position and 4 h in a supine position and tested the hypothesis of whether this repositioning protocol reduces the incidence of PUs in comparison with repositioning every 4 h. Interestingly, they found that the more frequent repositioning does not necessarily lead to fewer PUs, but their work still highlights the importance of repositioning as a routine clinical procedure for bed-bound patients.

To this date, the tolerance of skin to SPUs was not addressed by computer simulations allowing quantitative predictions of the risk of SPUs due to alternations in the moisture-related skin-support coefficient of friction (COF), and as related to repositioning. Independent to the effect of microclimate impact over the skin’s COF, these friction forces occur at acts of repositioning bed-bound patients at risk of PUs, which is a routine in geriatric, internal medicine, and long-term care hospital departments or nursing homes (Vanderwee et al., 2007). Herein, we investigated the mechanical interactions between the skin and a typical hospital mattress, depending on moisture-related changes in COF and skin stiffness, in the process of repositioning a bed-bound patient. We also intended to measure how the skin stiffness affects the effective stress distribution within the skin and subcutaneous tissues at the modeled region of interest (ROI), when repositioning.

## Materials and Methods

**Figure 1:**
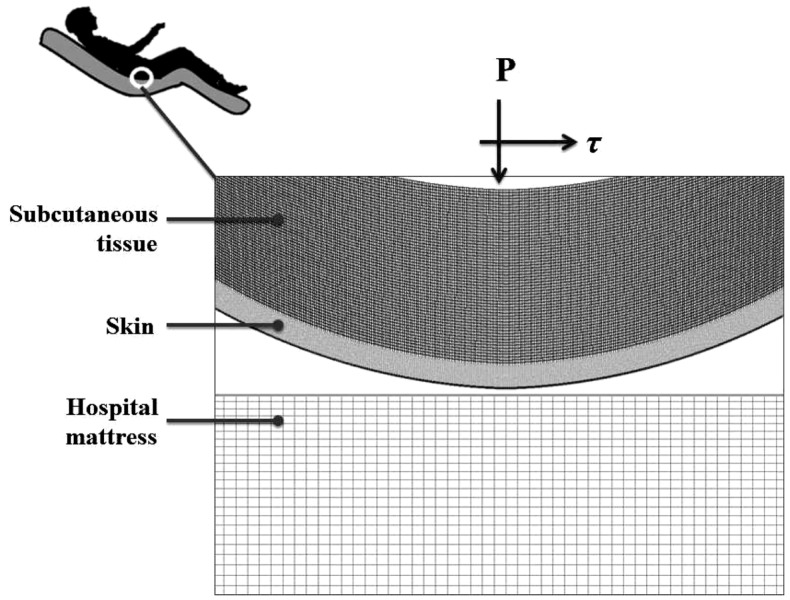
**The model of the skin in interaction of the hospital mattress**.

A finite element (FE) model has been developed, using the ADINA-AUI 8.8.1 software package. The purpose of this model was to assess biomechanical phenomena in a two-dimensional (2D) ROI representing the skin and subcutaneous tissues under a bony prominence of a bed-bound patient on a hospital mattress. The model was used to simulate the effects of skin-support COF changes due to changing wetness conditions (e.g., due to build-up of sweat or urine) on skin and subcutaneous loading, while considering the body load on the skin and subcutaneous tissues, as the ROI is pressured on the bed surface and moved across the bed. The physical dimensions of the model components (Figure 1) are specified in Table 1.

**Table 1:** **Values of the physical and mechanical properties, and of numerical characteristics used in this study**.

Parameter	Skin^a^	Subcutaneous tissue^a^	Hospital mattress^b^
Density (kg/m^3^)	1100	971	30
Poisson’s ratio (−)	0.49	0.48	0.3
Elastic modulus (kPa)	15.2/50/100	2	10
Thickness (mm)	2	15	50
Length (mm)	60	60	400
Number of elements (−)	8515	24300	20000

^a^Data were adopted from the literature (Todd and Thacker, 1994; Hendriks and Franklin, 2010; van Kuilenburg et al., 2013).

^b^Data were adopted from the literature (Linder-Ganz and Gefen, 2004).

The radius of curvature of the skin surface (Figure 1) was 180 mm.

Note that since we consider a case of a patient which is being moved on the support, such as during repositioning, the relevant mechanical property would be the instantaneous skin stiffness which is expressed here as the elastic modulus. The modeling was two-dimensional and hence all the thickness and length parameters referred to in this table are within the plane of Figure 1.

As PUs typically occur near bony prominences where there is always a curvature (of the bone surface), we defined a curved skin geometry which follows the simulated bone and subcutaneous tissue contours. The dimensions of the selected ROI could represent for example the ischial tuberosity region in the buttocks.

The computations were carried out in a plane stress analysis using the ADINA structure package with the default sparse solver. The model was solved with accuracy when the energy convergence criterion in the FE solver was set to be zero. The mesh was generated by quadrilateral elements. We refined the mesh of the skin layer (Figure 1), where effective stresses corresponding to high shear stresses were expected. Tied interfaces were defined between the skin-subcutaneous tissue components. A mesh validation convergence test was performed for the case in which maximal effective stresses were expected (that is, maximal COF = 0.8), which also guarantees accuracy for the remaining simulation cases. The working mesh (Figure 1) was chosen when the effective stresses received from two successively refined meshes differed by less than 2%. That was obtained when mesh densities exceeded ∼8000 elements at the skin layer; therefore we used this mesh density for all the analyses reported herein (Table 1). The physical and biomechanical properties of the ROI corresponded to skin contact modeling described in previous literature, and all relevant values for the model parameters are listed in Table 1 (Linder-Ganz and Gefen, 2004; van Kuilenburg et al., 2013).

The skin and subcutaneous tissues were modeled as linearly elastic isotropic nearly incompressible materials, which is suitable (as a first step) for assessing the instantaneous (stiffness) response of the skin and subcutaneous tissues to the relatively rapid movement during repositioning. Interestingly, a recent study supported this approach from a different perspective, arguing that skin-to-mattress contact analyses should not be addressed by viscoelastic parameters of the tissues in contact, due to the microclimate impact on skin hydration (Gerhardt et al., 2008a). The thickness of the skin and subcutaneous tissues were assumed here to be 2 and 15 mm, respectively, to represent an individual anatomy, but it is noted that anatomical variations in skin and subcutaneous tissue thicknesses across patients are expected in any real-world scenario (Table 1).

Based on large variations in literature regarding the elastic modulus of skin (as an effective material), we addressed a range of stiffness values in the domain of 10–100 kPa (Hendriks and Franklin, 2010) which can describe a difference between a more compliant young skin, and an aged stiffer skin (due to collagen-interlinking) or a process of a disease affecting collagen structure and interlinking such as type-2 diabetes, or variations in the same subject depending on exposure to chemicals of urine or feces (Gefen, 2011).

A pressure boundary condition was applied on the top edge of the model, in order to simulate the load over the ROI, generated by the relative body-weight imposed to the bony prominence and downwards to the outer tissue layers. Pressure under the bony prominence was estimated elsewhere (Holmes and Robb, 2006), and was set for all simulations at the level of 130 kPa which corresponds to a male with a normal body mass index. The hospital mattress (Figure 1) was constrained of any movement (translations or rotations) on the sides and the bottom. Constraining the mattress on the sides was needed in order to simulate the resistance to deformation from the lateral mattress parts outside the ROI (that is, which were not modeled).

It is well-established that the moisture-contents at the skin-support interface strongly influences the skin-support COF, with a drier environment allowing lower COF (Gerhardt et al., 2008a; Rotaru et al., 2013). Accordingly, and assuming a Coulomb friction model, the contact pair COF between the skin layers to the mattress was altered in the range of 0.2–0.8, to simulate low moisture levels (low COF; Rotaru et al., 2013) and up to a wet skin-support interface (high COF; Gerhardt et al., 2008a). Displacement was applied to the top edge of the model in a standard, lateral turning, assuming repositioning regime of 10 cm horizontal sliding along, and 1 cm toward (i.e., immersion into) the mattress. The aforementioned 130 kPa pressure represented the static weight-bearing of the patient, and the 1-cm displacement toward the mattress represented the additional loading applied by a caregiver to reposition the patient, where holding the patient steadily should induce some immersion of the patient’s body in bed.

The skin and subcutaneous stress data were always collected from the latest time-step of the simulations, that is, at the end-point of the displacement regime. In all simulations (Table 2), we measured stress levels in skin and subcutaneous tissue by extracting the maximal effective stress and shear stress from the midline of the model geometry, comprises of 20 elements of skin and 60 elements of subcutaneous tissues (Figure 1). For contact analysis, the interface effective stresses (that is, the von Mises stresses calculated using the pressure and shear at the skin-support interface), and the shear stresses were also calculated from the skin connecting layer.

**Table 2:** **Taguchi orthogonal array consisting of twelve simulations to assess the influence of change in contact pair COF (four levels, one being low, and four high) and skin stiffness (kPa) (three values, one being low, and three high) in the finite element model**.

Simulation	Contact pair COF	Elastic modulus of the skin layer (kPa)
1	0.2 (1)	15.2 (1)
2	0.2 (1)	50 (2)
3	0.2 (1)	100 (3)
4	0.4 (2)	15.2 (1)
5	0.4 (2)	50 (2)
6	0.4 (2)	100 (3)
7	0.6 (3)	15.2 (1)
8	0.6 (3)	50 (2)
9	0.6 (3)	100 (3)
10	0.8 (4)	15.2 (1)
11	0.8 (4)	50 (2)
12	0.8 (4)	100 (3)

Determined by the magnitude of maximal interface effective stress, we can confirm the relative importance of friction versus the skin stiffness. According to a Taguchi orthogonal array (Dar et al., 2002), the values prescribed in Table 2 eventually requires a total of 12 simulations: For each elastic modulus of the skin, the COF parameter varied by 50% from the reference contact pair COF of 0.4.

## Results

**Figure 2:**
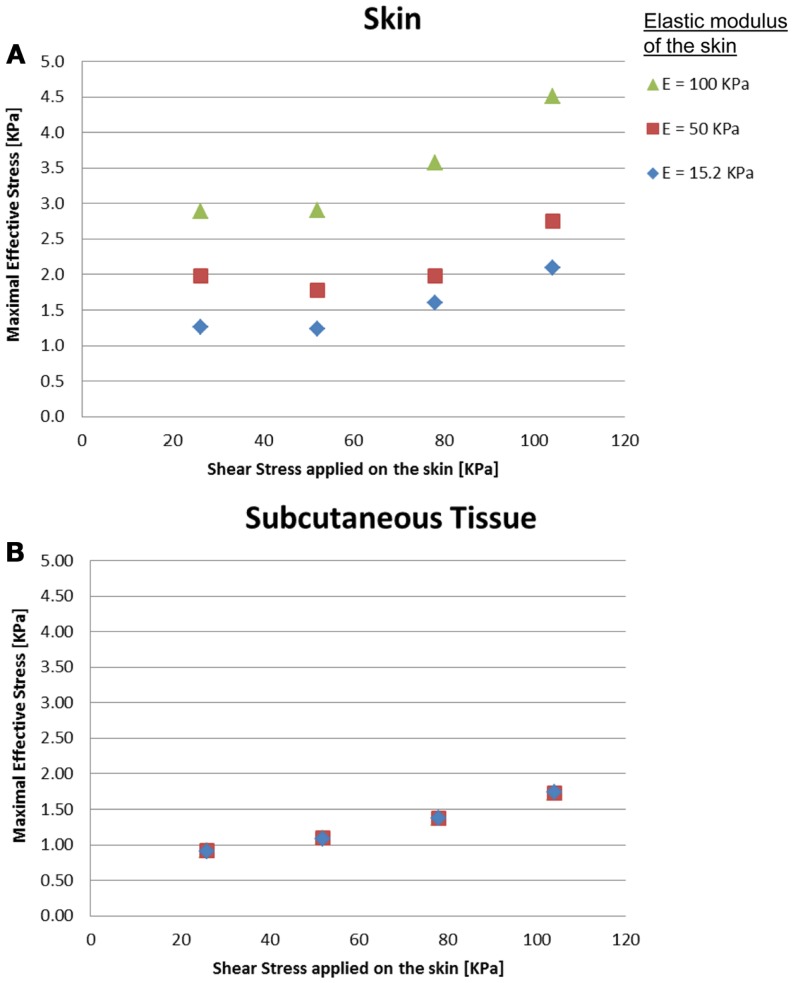
**Effects of a change in skin stiffness on the inner maximal effective stress: (A) skin stresses and (B) subcutaneous tissue stresses**. The stress analysis was time-dependent and the values referred to are the maximal stresses that occurred at the end of the maneuver of the simulated dragging of the body part over the mattress.

**Figure 3:**
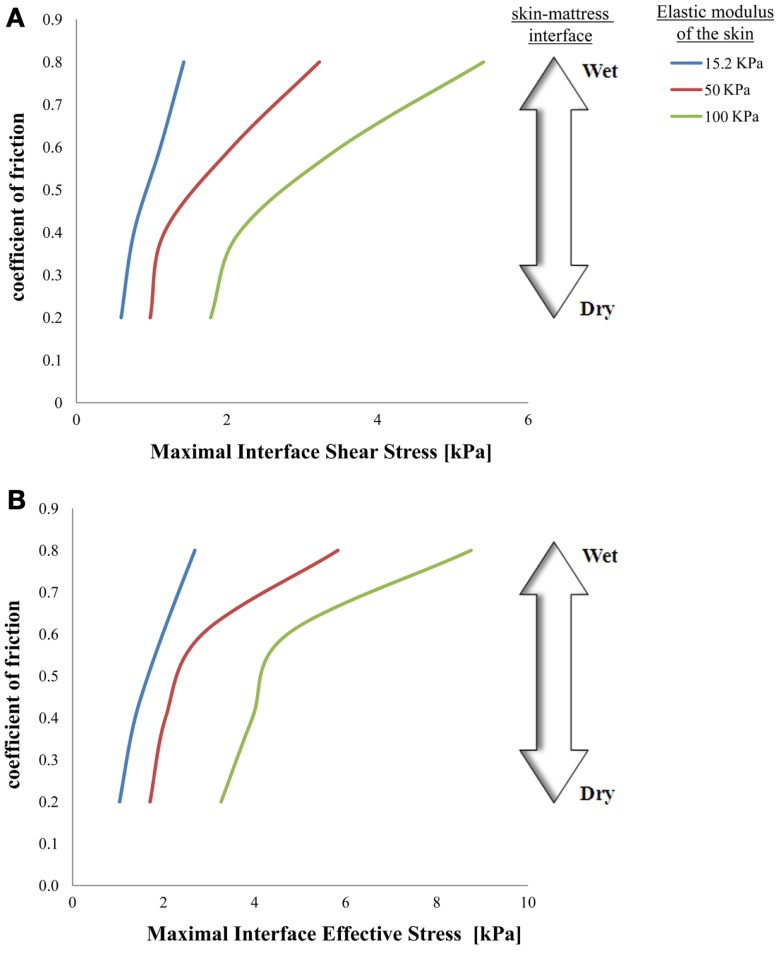
**Effects of a change in skin stiffness on maximal interface shear stress (A) and on maximal interface effective stress (B)**.

**Figure 4:**
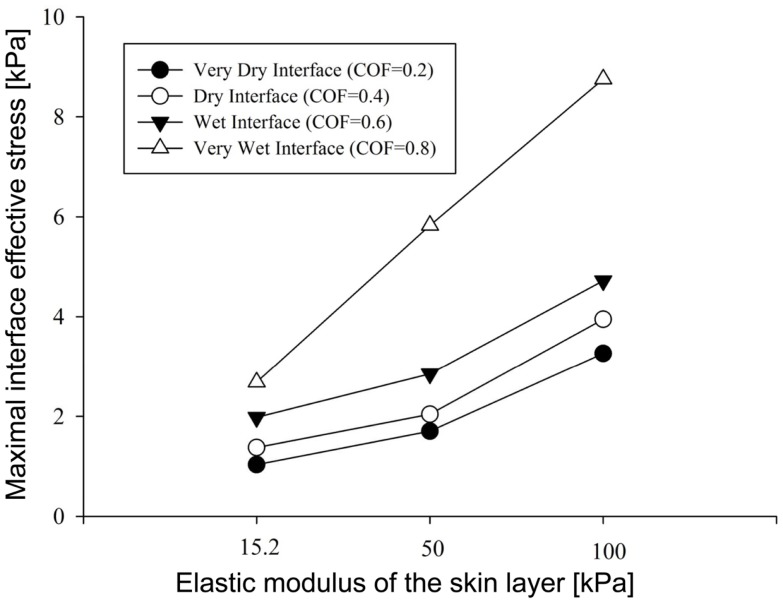
**Results of the factorial analysis: the influence of dry versus wet interface on maximal interface shear stress**.

The effects of a change in skin stiffness and/or moisture-contents-related COF on the skin internal and interface maximal effective stresses were investigated by varying the values of the elastic modulus of the skin and the contact pair COF in the simulations, respectively (Figure 2). The simulations indicated that the maximal effective stress in the skin increases as the skin-support COF rises, e.g., from 1.27, 1.98, and 2.89 kPa for skin stiffnesses of 15.2, 50, and 100 kPa when COF = 0.2, to a maximum of 2.09, 2.75, and 4.51 kPa for corresponding skin stiffnesses when COF = 0.6 (Figures 3 and 4).

Importantly, maximal effective stresses in the skin were substantially higher as the skin stiffness increased, with a rise of 55% when the elastic modulus of the skin was 100 kPa, compared to 15.2 kPa. In the subcutaneous tissues, on the other hand, the results show a mild rise of maximal effective stress, ranging from 0.91 to 1.75 kPa, with nearly no impact of the stiffness of the skin.

**Figure 5:**
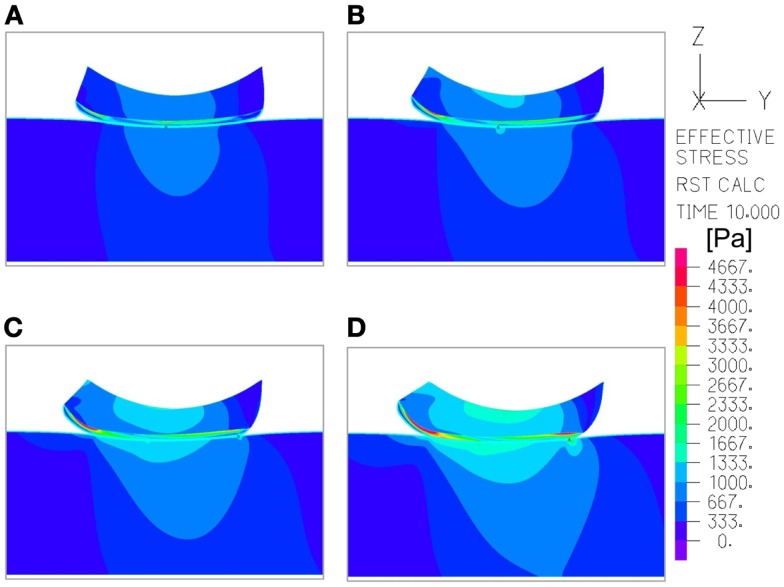
**An example of the distribution of effective stresses in the region of interest, at the end-point of the repositioning process in the simulations (*t* = 10 s), which depicts how the moisture-related skin-support coefficient of friction (COF) influences internal skin and subcutaneous stresses**. The skin and subcutaneous stress data were always collected from the latest time-step of the simulations, that is, at the end-point of the displacement regime, since tissue loads were maximal at that time point. In this example, the skin stiffness was 100 kPa and the COF varied as followed: **(A)** 0.2; **(B)** 0.4; **(C)** 0.6; **(D)** 0.8. The value range in the color bar was set to be from zero to a maximum of 4.5 kPa.

The simulations further indicated that when reaching full repositioning of 10 cm horizontal sliding and 1 cm toward the mattress, in weight-bearing of 130 kPa under the bony prominence, maximal interface shear stresses ranged from 0.59–1.43, 0.98–3.23, and 1.78–5.41 kPa for skin stiffnesses of 15.2, 50, and 100 kPa, respectively (Figures 2–5). The influence of the increase in the moisture-contents-related COF was more apparent when the skin was stiffer, reaching maximal interface shear stress of 5.41 kPa when the COF was 0.8 and the skin stiffness was 100 kPa. The maximal effective stress at the interface ranged from 1.0–2.7, 1.7–5.8, and 3.3–8.8 kPa for skin stiffnesses of 15.2, 50, and 100 kPa, respectively (Figure 3).

To summarize our present results: as could have been expected, the maximal effective stresses in the skin increase as the moisture-contents-related COF between the skin and the mattress rises. Interestingly however, the rise in stresses for a wet interface became more prominent when the skin tissue was stiffer – which represented aging or diabetes (Figure 4). This important finding demonstrates how the aged/diabetic skin is more fragile than a young-adult skin when repositioning in a moist environment.

## Discussion

Repositioning bed-bound patients, such as patients in geriatric, internal medicine, long-term care, and other hospital departments as well as nursing homes is a day-to-day routine. Often patients suffer incontinence problems, or are sweating in bed (e.g., due to fever), which induces a moist environment to the skin. Most clinicians are aware, based on experience and intuition that these conditions make the skin more fragile and suspected to SPUs, but the biomechanics of the routine care procedure of repositioning was never addressed in the literature, and, specifically, no modeling was done in this regard. The loading of skin during a repositioning maneuver, in the context of SPUs, was therefore examined in the simulations presented here. Recent studies stress the fact that microclimate conditions are related to skin tolerance problems that may lead to SPUs (Gefen, 2011; Yusuf et al., 2013). Herein, we focused on the roles of friction and skin stiffness, and the interactions between these factors, via computational modeling, to assess the impact of wetness changes at the skin-to-bed interface. In particular, since it is widely reported that older bed-bound patients’ skin or the skin of individuals with type-2 diabetes have a stiffer behavior than that of younger patients (Edwards and Marks, 1995; Sopher and Gefen, 2011; Schulze et al., 2012; van Kuilenburg et al., 2013), it seemed reasonable to address this contact problem focusing on the interactions between skin stiffness, moisture-contents-related skin-support COF, and interface/internal skin loading.

Repositioning of bed-bound patients must involve the rubbing of their skin against the surface of the hospital mattress (and the clothing as well). These friction and pressure affronts may lead to shearing injury and raise the risk of SPUs (Gefen, 2011; Lahmann and Kottner, 2011). In our simulations, during repositioning, the wetter the interface between the skin under the IT and the hospital mattress was (which increases the COF) – higher values of effective stress occurred within the skin layer (Figures 3–5). Importantly, this rise in stresses became more prominent when the skin tissue was stiffer – which represented aging or diabetes (Figure 4). We hence demonstrated using the modeling, for the first time in the literature, that the aged/diabetic skin is more fragile than a young-adult skin when repositioning in a moist environment, which certainly agrees with clinical experience but was lacking scientific evidence.

Based on the above findings, one can think of better controlling the skin-support friction by either reducing the COF (by keeping the skin dry, or by using creams to lower the COF) or by reducing the stiffness of the skin in susceptible areas for SPUs, for example by using lubricants that are absorbed into the skin layers. Lubricants may promote softening of the skin (Adams et al., 2007), and can have beneficial effects over the skin contact problem, as elucidated elsewhere (Schulze et al., 2012) and also as suggested in this study. Nevertheless, the COF increases upon moist skin, due to wider contact area (Gerhardt et al., 2008a), which can result in unfavorable outcomes while enduring loads from underlying layers in bony prominences and to external forces (Yusuf et al., 2013). Also, if the skin remains wet or moist due to microclimate conditions (Gefen, 2011), over-lubrication or perhaps exposure to urine or feces increases the skin-support COF and consequently reduces the tolerance of skin to SPUs (Gerhardt et al., 2008a; Gefen, 2011; Rotaru et al., 2013).

In our simulations we presented the interfacial skin-support shear stress on top of internal skin stresses, as it can represent the ability of the skin layer to absorb the repositioning displacements rather than transferring the shearing loads into the underlying layers (Akins et al., 2011). Our results stand within the range of maximal interface shear stresses contributing to SPUs which were presented elsewhere (Shang and Bai, 2012).

Since we considered a case of a patient who is being moved on the support, during repositioning, the relevant mechanical property was the instantaneous skin stiffness which is expressed here as the skin’s elastic modulus (Table 1). Future modeling can consider viscoelastic constitutive laws for skin and subcutaneous tissues, but this will only become important if one attempts to quantify the effects of the speed of the repositioning maneuver on tissue stresses and the corresponding risk for tissue failure. We did not analyze the effect of the horizontal velocity of the repositioning, given that this would be somewhat theoretical, and rather difficult to control in a clinical environment (that is, in a real-world scenario). Nevertheless, it may be worthwhile to explore the influence of this velocity in the future, in the context of refining guidelines, to instruct caregivers to pay attention to, e.g., how delicate they are in performing these maneuvers.

This is the first FE model ever to investigate repositioning and the related risk for PUs. Though models can always be made more complicated to represent, e.g., a more detailed anatomy, more complex tissue mechanical behaviors, and interactions, more refined representation of the support, clothing and bed sheets, etc. one needs to also consider that these will add parameters and interactions in the model. A general philosophy when approaching a problem for the first time would be to start with a relatively simple model which includes just a limited set of parameters, in order to attempt and isolate the most important trends of effects. Clearly further modeling work can build upon the current study, and incorporate more of the aforementioned phenomena, but while keeping in mind that in the context of microclimate, mechanical interactions in the wet skin and skin fragility with age and disease, there are vast gaps in empirical information that should be addressed first. Hence, while appreciating that the present modeling was relatively simple (2D, linear elastic), it highlights – for the first time – important topics that are highly relevant to the day-to-day routine of many hospital and nursing home settings, and provides explanations and insights that were not reported previously.

Although the skin stiffness varied in our simulations, the displacement regime and the perpendicular pressure were kept constant. In Figure 3, we assess the effect of friction and the skin stiffness, over the interfacial shear stresses: we found that skin stiffness has adequate impact over the maximal interfacial shear stresses as a result of the repositioning regime. Increase in the value of the COF results in increase of the maximal interface shear stress (Figures 3A and 4), the maximal interface effective stress (Figure 3B), and over the distribution of effective stresses within the skin layer and subcutaneous tissue (Figure 5). Thus, repositioning an elderly bed-bound patient with his/her aged skin, i.e., stiffer skin layer, in wet/moist conditions, increases the risk of SPUs while repositioning, which is also consistent with the factorial analysis in Figure 4, which clearly demonstrates that wet interfaces have a more substantial impact over the maximal interface effective stresses.

The repositioning scenario investigated here should be addressed in the future by more detailed computational models, e.g., also considering the sheets/fabrics and their materials and textures. By taking these types of external factors into account, it would be possible to achieve deeper understanding of the pathomechanics and perhaps even evaluate or rate hospital clothing materials, bed sheets, and other elements that interact with the skin of patients at risk for PUs.

Our present study emphasizes the importance of taking a preventative action of making sure the skin-to-mattress interface remains dry (that is, free or sweat or urine), prior to repositioning patients. Surprisingly, the rise in stresses for a wet interface became more prominent when the skin tissue was stiffer. While the other present results could perhaps be expected or are more intuitive, this one could not be foreseen, and have practical implications with respect to care of the elderly and diabetic populations. The simulations therefore highlighted the risk in repositioning elderly or diabetic patients with stiffer skin properties in a wet environment, which provides scientific evidence to support clinical practice in this regard. The modeling used herein can now be extended to test effects of different moisturizers, creams, lubricants, or possibly other interventions at the skin-support interface for testing their potential in protecting the skin from SPUs in a standard, objective, and quantitative manner.

## Conflict of Interest Statement

The authors declare that the research was conducted in the absence of any commercial or financial relationships that could be construed as a potential conflict of interest.
